# Supplementary material for: Iterative improvement in the automatic modular design of robot swarms
Source: PeerJ Comput Sci. 2020 Dec 7;6:e322. doi: 10.7717/peerj-cs.322 (PMC7924708; doi:10.7717/peerj-cs.322)
Supplement: Supplemental Information 3 [file peerj-cs-06-322-s003.zip › argos3/doc/api/standalone/a00464_source.html]

ARGoS: plugins/robots/foot-bot/simulator/footbot\_entity.cpp Source File


- Main Page
- Related Pages
- Namespaces
- Classes
- Files

- File List
- File Members

# plugins/robots/foot-bot/simulator/footbot\_entity.cpp

Go to the documentation of this file.

```
00001 
00007 #include "footbot_entity.h"
00008 
00009 #include <argos3/core/utility/math/matrix/rotationmatrix3.h>
00010 #include <argos3/core/simulator/space/space.h>
00011 #include <argos3/core/simulator/entity/controllable_entity.h>
00012 #include <argos3/core/simulator/entity/embodied_entity.h>
00013 #include <argos3/plugins/simulator/entities/rab_equipped_entity.h>
00014 #include <argos3/plugins/simulator/entities/gripper_equipped_entity.h>
00015 #include <argos3/plugins/simulator/entities/ground_sensor_equipped_entity.h>
00016 #include <argos3/plugins/simulator/entities/led_equipped_entity.h>
00017 #include <argos3/plugins/simulator/entities/light_sensor_equipped_entity.h>
00018 #include <argos3/plugins/simulator/entities/omnidirectional_camera_equipped_entity.h>
00019 #include <argos3/plugins/simulator/entities/perspective_camera_equipped_entity.h>
00020 #include <argos3/plugins/simulator/entities/proximity_sensor_equipped_entity.h>
00021 #include <argos3/plugins/simulator/entities/wifi_equipped_entity.h>
00022 #include "footbot_distance_scanner_equipped_entity.h"
00023 #include "footbot_turret_entity.h"
00024 
00025 namespace argos {
00026 
00027    /****************************************/
00028    /****************************************/
00029 
00030    static const Real BODY_RADIUS                = 0.085036758f;
00031    static const Real BODY_HEIGHT                = 0.146899733f;
00032 
00033    static const Real LED_RING_RADIUS            = BODY_RADIUS + 0.005;
00034 
00035    static const Real INTERWHEEL_DISTANCE        = 0.14f;
00036    static const Real HALF_INTERWHEEL_DISTANCE   = INTERWHEEL_DISTANCE * 0.5f;
00037    static const Real WHEEL_RADIUS               = 0.029112741f;
00038 
00039    static const Real PROXIMITY_SENSOR_RING_ELEVATION       = 0.06f;
00040    static const Real PROXIMITY_SENSOR_RING_RADIUS          = BODY_RADIUS;
00041    static const CRadians PROXIMITY_SENSOR_RING_START_ANGLE = CRadians((ARGOS_PI / 12.0f) * 0.5f);
00042    static const Real PROXIMITY_SENSOR_RING_RANGE           = 0.1f;
00043 
00044    static const Real LED_RING_ELEVATION         = 0.085f;
00045    static const Real RAB_ELEVATION              = 0.1f;
00046    static const Real BEACON_ELEVATION           = 0.174249733f;
00047 
00048    static const Real GRIPPER_ELEVATION          = LED_RING_ELEVATION;
00049 
00050    static const CRadians LED_ANGLE_SLICE        = CRadians(ARGOS_PI / 6.0);
00051    static const CRadians HALF_LED_ANGLE_SLICE   = LED_ANGLE_SLICE * 0.5f;
00052 
00053    static const Real OMNIDIRECTIONAL_CAMERA_ELEVATION = 0.288699733f;
00054 
00055    /****************************************/
00056    /****************************************/
00057 
00058    CFootBotEntity::CFootBotEntity() :
00059       CComposableEntity(NULL),
00060       m_pcControllableEntity(NULL),
00061       m_pcDistanceScannerEquippedEntity(NULL),
00062       m_pcTurretEntity(NULL),
00063       m_pcEmbodiedEntity(NULL),
00064       m_pcGripperEquippedEntity(NULL),
00065       m_pcGroundSensorEquippedEntity(NULL),
00066       m_pcLEDEquippedEntity(NULL),
00067       m_pcLightSensorEquippedEntity(NULL),
00068       m_pcOmnidirectionalCameraEquippedEntity(NULL),
00069       m_pcPerspectiveCameraEquippedEntity(NULL),
00070       m_pcProximitySensorEquippedEntity(NULL),
00071       m_pcRABEquippedEntity(NULL),
00072       m_pcWheeledEntity(NULL),
00073       m_pcWiFiEquippedEntity(NULL) {
00074    }
00075 
00076    /****************************************/
00077    /****************************************/
00078 
00079    CFootBotEntity::CFootBotEntity(const std::string& str_id,
00080                                   const std::string& str_controller_id,
00081                                   const CVector3& c_position,
00082                                   const CQuaternion& c_orientation,
00083                                   Real f_rab_range,
00084                                   size_t un_rab_data_size,
00085                                   const CRadians& c_omnicam_aperture,
00086                                   bool b_perspcam_front,
00087                                   const CRadians& c_perspcam_aperture,
00088                                   Real f_perspcam_focal_length,
00089                                   Real f_perspcam_range) :
00090       CComposableEntity(NULL, str_id),
00091       m_pcControllableEntity(NULL),
00092       m_pcDistanceScannerEquippedEntity(NULL),
00093       m_pcTurretEntity(NULL),
00094       m_pcEmbodiedEntity(NULL),
00095       m_pcGripperEquippedEntity(NULL),
00096       m_pcGroundSensorEquippedEntity(NULL),
00097       m_pcLEDEquippedEntity(NULL),
00098       m_pcLightSensorEquippedEntity(NULL),
00099       m_pcOmnidirectionalCameraEquippedEntity(NULL),
00100       m_pcPerspectiveCameraEquippedEntity(NULL),
00101       m_pcProximitySensorEquippedEntity(NULL),
00102       m_pcRABEquippedEntity(NULL),
00103       m_pcWheeledEntity(NULL),
00104       m_pcWiFiEquippedEntity(NULL) {
00105       try {
00106          /*
00107           * Create and init components
00108           */
00109          /*
00110           * Embodied entity
00111           * Better to put this first, because many other entities need this one
00112           */
00113          m_pcEmbodiedEntity = new CEmbodiedEntity(this, "body_0", c_position, c_orientation);
00114          AddComponent(*m_pcEmbodiedEntity);
00115          SAnchor& cTurretAnchor = m_pcEmbodiedEntity->AddAnchor("turret");
00116          CQuaternion cPerspCamOrient(b_perspcam_front ? CRadians::ZERO : -CRadians::PI_OVER_TWO,
00117                                      CVector3::Y);
00118          SAnchor& cPerspCamAnchor = m_pcEmbodiedEntity->AddAnchor("perspective_camera",
00119                                                                   CVector3(BODY_RADIUS, 0.0, BEACON_ELEVATION),
00120                                                                   cPerspCamOrient);
00121          /* Wheeled entity and wheel positions (left, right) */
00122          m_pcWheeledEntity = new CWheeledEntity(this, "wheels_0", 2);
00123          AddComponent(*m_pcWheeledEntity);
00124          m_pcWheeledEntity->SetWheel(0, CVector3(0.0f,  HALF_INTERWHEEL_DISTANCE, 0.0f), WHEEL_RADIUS);
00125          m_pcWheeledEntity->SetWheel(1, CVector3(0.0f, -HALF_INTERWHEEL_DISTANCE, 0.0f), WHEEL_RADIUS);
00126          /* LED equipped entity, with LEDs [0-11] and beacon [12] */
00127          m_pcLEDEquippedEntity = new CLEDEquippedEntity(this, "leds_0");
00128          AddComponent(*m_pcLEDEquippedEntity);
00129          m_pcLEDEquippedEntity->AddLEDRing(
00130             CVector3(0.0f, 0.0f, LED_RING_ELEVATION),
00131             LED_RING_RADIUS,
00132             HALF_LED_ANGLE_SLICE,
00133             12,
00134             cTurretAnchor);
00135          m_pcLEDEquippedEntity->AddLED(
00136             CVector3(0.0f, 0.0f, BEACON_ELEVATION),
00137             cTurretAnchor);
00138          /* Proximity sensor equipped entity */
00139          m_pcProximitySensorEquippedEntity =
00140             new CProximitySensorEquippedEntity(this, "proximity_0");
00141          AddComponent(*m_pcProximitySensorEquippedEntity);
00142          m_pcProximitySensorEquippedEntity->AddSensorRing(
00143             CVector3(0.0f, 0.0f, PROXIMITY_SENSOR_RING_ELEVATION),
00144             PROXIMITY_SENSOR_RING_RADIUS,
00145             PROXIMITY_SENSOR_RING_START_ANGLE,
00146             PROXIMITY_SENSOR_RING_RANGE,
00147             24,
00148             m_pcEmbodiedEntity->GetOriginAnchor());
00149          /* Light sensor equipped entity */
00150          m_pcLightSensorEquippedEntity =
00151             new CLightSensorEquippedEntity(this, "light_0");
00152          AddComponent(*m_pcLightSensorEquippedEntity);
00153          m_pcLightSensorEquippedEntity->AddSensorRing(
00154             CVector3(0.0f, 0.0f, PROXIMITY_SENSOR_RING_ELEVATION),
00155             PROXIMITY_SENSOR_RING_RADIUS,
00156             PROXIMITY_SENSOR_RING_START_ANGLE,
00157             PROXIMITY_SENSOR_RING_RANGE,
00158             24,
00159             m_pcEmbodiedEntity->GetOriginAnchor());
00160          /* Gripper equipped entity */
00161          m_pcGripperEquippedEntity =
00162             new CGripperEquippedEntity(this,
00163                                        "gripper_0",
00164                                        CVector3(BODY_RADIUS, 0.0f, GRIPPER_ELEVATION),
00165                                        CVector3::X);
00166          AddComponent(*m_pcGripperEquippedEntity);
00167          /* Ground sensor equipped entity */
00168          m_pcGroundSensorEquippedEntity =
00169             new CGroundSensorEquippedEntity(this, "ground_0");
00170          AddComponent(*m_pcGroundSensorEquippedEntity);
00171          m_pcGroundSensorEquippedEntity->AddSensor(CVector2(0.063, 0.0116),
00172                                                    CGroundSensorEquippedEntity::TYPE_GRAYSCALE,
00173                                                    m_pcEmbodiedEntity->GetOriginAnchor());
00174          m_pcGroundSensorEquippedEntity->AddSensor(CVector2(-0.063, 0.0116),
00175                                                    CGroundSensorEquippedEntity::TYPE_GRAYSCALE,
00176                                                    m_pcEmbodiedEntity->GetOriginAnchor());
00177          m_pcGroundSensorEquippedEntity->AddSensor(CVector2(-0.063, -0.0116),
00178                                                    CGroundSensorEquippedEntity::TYPE_GRAYSCALE,
00179                                                    m_pcEmbodiedEntity->GetOriginAnchor());
00180          m_pcGroundSensorEquippedEntity->AddSensor(CVector2(0.063, -0.0116),
00181                                                    CGroundSensorEquippedEntity::TYPE_GRAYSCALE,
00182                                                    m_pcEmbodiedEntity->GetOriginAnchor());
00183          m_pcGroundSensorEquippedEntity->AddSensor(CVector2(0.08, 0.0),
00184                                                    CGroundSensorEquippedEntity::TYPE_BLACK_WHITE,
00185                                                    m_pcEmbodiedEntity->GetOriginAnchor());
00186          m_pcGroundSensorEquippedEntity->AddSensor(CVector2(0.042, 0.065),
00187                                                    CGroundSensorEquippedEntity::TYPE_BLACK_WHITE,
00188                                                    m_pcEmbodiedEntity->GetOriginAnchor());
00189          m_pcGroundSensorEquippedEntity->AddSensor(CVector2(0.0, 0.08),
00190                                                    CGroundSensorEquippedEntity::TYPE_BLACK_WHITE,
00191                                                    m_pcEmbodiedEntity->GetOriginAnchor());
00192          m_pcGroundSensorEquippedEntity->AddSensor(CVector2(-0.042, 0.065),
00193                                                    CGroundSensorEquippedEntity::TYPE_BLACK_WHITE,
00194                                                    m_pcEmbodiedEntity->GetOriginAnchor());
00195          m_pcGroundSensorEquippedEntity->AddSensor(CVector2(-0.08, 0.0),
00196                                                    CGroundSensorEquippedEntity::TYPE_BLACK_WHITE,
00197                                                    m_pcEmbodiedEntity->GetOriginAnchor());
00198          m_pcGroundSensorEquippedEntity->AddSensor(CVector2(-0.042, -0.065),
00199                                                    CGroundSensorEquippedEntity::TYPE_BLACK_WHITE,
00200                                                    m_pcEmbodiedEntity->GetOriginAnchor());
00201          m_pcGroundSensorEquippedEntity->AddSensor(CVector2(0.0, -0.08),
00202                                                    CGroundSensorEquippedEntity::TYPE_BLACK_WHITE,
00203                                                    m_pcEmbodiedEntity->GetOriginAnchor());
00204          m_pcGroundSensorEquippedEntity->AddSensor(CVector2(0.042, -0.065),
00205                                                    CGroundSensorEquippedEntity::TYPE_BLACK_WHITE,
00206                                                    m_pcEmbodiedEntity->GetOriginAnchor());
00207          /* Distance scanner */
00208          m_pcDistanceScannerEquippedEntity =
00209             new CFootBotDistanceScannerEquippedEntity(this, "distance_scanner_0");
00210          AddComponent(*m_pcDistanceScannerEquippedEntity);
00211          /* RAB equipped entity */
00212          m_pcRABEquippedEntity =
00213             new CRABEquippedEntity(this,
00214                                    "rab_0",
00215                                    un_rab_data_size,
00216                                    f_rab_range,
00217                                    m_pcEmbodiedEntity->GetOriginAnchor(),
00218                                    *m_pcEmbodiedEntity,
00219                                    CVector3(0.0f, 0.0f, RAB_ELEVATION));
00220          AddComponent(*m_pcRABEquippedEntity);
00221          /* Omnidirectional camera equipped entity */
00222          m_pcOmnidirectionalCameraEquippedEntity =
00223             new COmnidirectionalCameraEquippedEntity(this,
00224                                                      "omnidirectional_camera_0",
00225                                                      c_omnicam_aperture,
00226                                                      CVector3(0.0f,
00227                                                               0.0f,
00228                                                               OMNIDIRECTIONAL_CAMERA_ELEVATION));
00229          AddComponent(*m_pcOmnidirectionalCameraEquippedEntity);
00230          /* Perspective camera equipped entity */
00231          m_pcPerspectiveCameraEquippedEntity =
00232             new CPerspectiveCameraEquippedEntity(this,
00233                                                  "perspective_camera_0",
00234                                                  c_perspcam_aperture,
00235                                                  f_perspcam_focal_length,
00236                                                  f_perspcam_range,
00237                                                  640, 480,
00238                                                  cPerspCamAnchor);
00239          AddComponent(*m_pcPerspectiveCameraEquippedEntity);
00240          /* Turret equipped entity */
00241          m_pcTurretEntity = new CFootBotTurretEntity(this, "turret_0", cTurretAnchor);
00242          AddComponent(*m_pcTurretEntity);
00243          /* WiFi equipped entity */
00244          m_pcWiFiEquippedEntity = new CWiFiEquippedEntity(this, "wifi_0");
00245          AddComponent(*m_pcWiFiEquippedEntity);
00246          /* Controllable entity
00247             It must be the last one, for actuators/sensors to link to composing entities correctly */
00248          m_pcControllableEntity = new CControllableEntity(this, "controller_0");
00249          AddComponent(*m_pcControllableEntity);
00250          m_pcControllableEntity->SetController(str_controller_id);
00251          /* Update components */
00252          UpdateComponents();
00253       }
00254       catch(CARGoSException& ex) {
00255          THROW_ARGOSEXCEPTION_NESTED("Failed to initialize entity \"" << GetId() << "\".", ex);
00256       }
00257    }
00258 
00259    /****************************************/
00260    /****************************************/
00261 
00262    void CFootBotEntity::Init(TConfigurationNode& t_tree) {
00263       try {
00264          /*
00265           * Init parent
00266           */
00267          CComposableEntity::Init(t_tree);
00268          /*
00269           * Create and init components
00270           */
00271          /*
00272           * Embodied entity
00273           * Better to put this first, because many other entities need this one
00274           */
00275          m_pcEmbodiedEntity = new CEmbodiedEntity(this);
00276          AddComponent(*m_pcEmbodiedEntity);
00277          m_pcEmbodiedEntity->Init(GetNode(t_tree, "body"));
00278          SAnchor& cTurretAnchor = m_pcEmbodiedEntity->AddAnchor("turret");
00279          /* Wheeled entity and wheel positions (left, right) */
00280          m_pcWheeledEntity = new CWheeledEntity(this, "wheels_0", 2);
00281          AddComponent(*m_pcWheeledEntity);
00282          m_pcWheeledEntity->SetWheel(0, CVector3(0.0f,  HALF_INTERWHEEL_DISTANCE, 0.0f), WHEEL_RADIUS);
00283          m_pcWheeledEntity->SetWheel(1, CVector3(0.0f, -HALF_INTERWHEEL_DISTANCE, 0.0f), WHEEL_RADIUS);
00284          /* LED equipped entity, with LEDs [0-11] and beacon [12] */
00285          m_pcLEDEquippedEntity = new CLEDEquippedEntity(this, "leds_0");
00286          AddComponent(*m_pcLEDEquippedEntity);
00287          m_pcLEDEquippedEntity->AddLEDRing(
00288             CVector3(0.0f, 0.0f, LED_RING_ELEVATION),
00289             LED_RING_RADIUS,
00290             HALF_LED_ANGLE_SLICE,
00291             12,
00292             cTurretAnchor);
00293          m_pcLEDEquippedEntity->AddLED(
00294             CVector3(0.0f, 0.0f, BEACON_ELEVATION),
00295             cTurretAnchor);
00296          /* Proximity sensor equipped entity */
00297          m_pcProximitySensorEquippedEntity =
00298             new CProximitySensorEquippedEntity(this, "proximity_0");
00299          AddComponent(*m_pcProximitySensorEquippedEntity);
00300          m_pcProximitySensorEquippedEntity->AddSensorRing(
00301             CVector3(0.0f, 0.0f, PROXIMITY_SENSOR_RING_ELEVATION),
00302             PROXIMITY_SENSOR_RING_RADIUS,
00303             PROXIMITY_SENSOR_RING_START_ANGLE,
00304             PROXIMITY_SENSOR_RING_RANGE,
00305             24,
00306             m_pcEmbodiedEntity->GetOriginAnchor());
00307          /* Light sensor equipped entity */
00308          m_pcLightSensorEquippedEntity =
00309             new CLightSensorEquippedEntity(this, "light_0");
00310          AddComponent(*m_pcLightSensorEquippedEntity);
00311          m_pcLightSensorEquippedEntity->AddSensorRing(
00312             CVector3(0.0f, 0.0f, PROXIMITY_SENSOR_RING_ELEVATION),
00313             PROXIMITY_SENSOR_RING_RADIUS,
00314             PROXIMITY_SENSOR_RING_START_ANGLE,
00315             PROXIMITY_SENSOR_RING_RANGE,
00316             24,
00317             m_pcEmbodiedEntity->GetOriginAnchor());
00318          /* Gripper equipped entity */
00319          m_pcGripperEquippedEntity =
00320             new CGripperEquippedEntity(this,
00321                                        "gripper_0",
00322                                        CVector3(BODY_RADIUS, 0.0f, GRIPPER_ELEVATION),
00323                                        CVector3::X);
00324          AddComponent(*m_pcGripperEquippedEntity);
00325          /* Ground sensor equipped entity */
00326          m_pcGroundSensorEquippedEntity =
00327             new CGroundSensorEquippedEntity(this, "ground_0");
00328          AddComponent(*m_pcGroundSensorEquippedEntity);
00329          m_pcGroundSensorEquippedEntity->AddSensor(CVector2(0.063, 0.0116),
00330                                                    CGroundSensorEquippedEntity::TYPE_GRAYSCALE,
00331                                                    m_pcEmbodiedEntity->GetOriginAnchor());
00332          m_pcGroundSensorEquippedEntity->AddSensor(CVector2(-0.063, 0.0116),
00333                                                    CGroundSensorEquippedEntity::TYPE_GRAYSCALE,
00334                                                    m_pcEmbodiedEntity->GetOriginAnchor());
00335          m_pcGroundSensorEquippedEntity->AddSensor(CVector2(-0.063, -0.0116),
00336                                                    CGroundSensorEquippedEntity::TYPE_GRAYSCALE,
00337                                                    m_pcEmbodiedEntity->GetOriginAnchor());
00338          m_pcGroundSensorEquippedEntity->AddSensor(CVector2(0.063, -0.0116),
00339                                                    CGroundSensorEquippedEntity::TYPE_GRAYSCALE,
00340                                                    m_pcEmbodiedEntity->GetOriginAnchor());
00341          m_pcGroundSensorEquippedEntity->AddSensor(CVector2(0.08, 0.0),
00342                                                    CGroundSensorEquippedEntity::TYPE_BLACK_WHITE,
00343                                                    m_pcEmbodiedEntity->GetOriginAnchor());
00344          m_pcGroundSensorEquippedEntity->AddSensor(CVector2(0.042, 0.065),
00345                                                    CGroundSensorEquippedEntity::TYPE_BLACK_WHITE,
00346                                                    m_pcEmbodiedEntity->GetOriginAnchor());
00347          m_pcGroundSensorEquippedEntity->AddSensor(CVector2(0.0, 0.08),
00348                                                    CGroundSensorEquippedEntity::TYPE_BLACK_WHITE,
00349                                                    m_pcEmbodiedEntity->GetOriginAnchor());
00350          m_pcGroundSensorEquippedEntity->AddSensor(CVector2(-0.042, 0.065),
00351                                                    CGroundSensorEquippedEntity::TYPE_BLACK_WHITE,
00352                                                    m_pcEmbodiedEntity->GetOriginAnchor());
00353          m_pcGroundSensorEquippedEntity->AddSensor(CVector2(-0.08, 0.0),
00354                                                    CGroundSensorEquippedEntity::TYPE_BLACK_WHITE,
00355                                                    m_pcEmbodiedEntity->GetOriginAnchor());
00356          m_pcGroundSensorEquippedEntity->AddSensor(CVector2(-0.042, -0.065),
00357                                                    CGroundSensorEquippedEntity::TYPE_BLACK_WHITE,
00358                                                    m_pcEmbodiedEntity->GetOriginAnchor());
00359          m_pcGroundSensorEquippedEntity->AddSensor(CVector2(0.0, -0.08),
00360                                                    CGroundSensorEquippedEntity::TYPE_BLACK_WHITE,
00361                                                    m_pcEmbodiedEntity->GetOriginAnchor());
00362          m_pcGroundSensorEquippedEntity->AddSensor(CVector2(0.042, -0.065),
00363                                                    CGroundSensorEquippedEntity::TYPE_BLACK_WHITE,
00364                                                    m_pcEmbodiedEntity->GetOriginAnchor());
00365          /* Distance scanner */
00366          m_pcDistanceScannerEquippedEntity =
00367             new CFootBotDistanceScannerEquippedEntity(this, "distance_scanner_0");
00368          AddComponent(*m_pcDistanceScannerEquippedEntity);
00369          /* RAB equipped entity */
00370          Real fRange = 3.0f;
00371          GetNodeAttributeOrDefault(t_tree, "rab_range", fRange, fRange);
00372          UInt32 unDataSize = 10;
00373          GetNodeAttributeOrDefault(t_tree, "rab_data_size", unDataSize, unDataSize);
00374          m_pcRABEquippedEntity =
00375             new CRABEquippedEntity(this,
00376                                    "rab_0",
00377                                    unDataSize,
00378                                    fRange,
00379                                    m_pcEmbodiedEntity->GetOriginAnchor(),
00380                                    *m_pcEmbodiedEntity,
00381                                    CVector3(0.0f, 0.0f, RAB_ELEVATION));
00382          AddComponent(*m_pcRABEquippedEntity);
00383          /* Omnidirectional camera equipped entity */
00384          CDegrees cAperture(70.0f);
00385          GetNodeAttributeOrDefault(t_tree, "omnidirectional_camera_aperture", cAperture, cAperture);
00386          m_pcOmnidirectionalCameraEquippedEntity =
00387             new COmnidirectionalCameraEquippedEntity(this,
00388                                                      "omnidirectional_camera_0",
00389                                                      ToRadians(cAperture),
00390                                                      CVector3(0.0f,
00391                                                               0.0f,
00392                                                               OMNIDIRECTIONAL_CAMERA_ELEVATION));
00393          AddComponent(*m_pcOmnidirectionalCameraEquippedEntity);
00394          /* Perspective camera equipped entity */
00395          bool bPerspCamFront = true;
00396          GetNodeAttributeOrDefault(t_tree, "perspective_camera_front", bPerspCamFront, bPerspCamFront);
00397          Real fPerspCamFocalLength = 0.035;
00398          GetNodeAttributeOrDefault(t_tree, "perspective_camera_focal_length", fPerspCamFocalLength, fPerspCamFocalLength);
00399          Real fPerspCamRange = 2.0;
00400          GetNodeAttributeOrDefault(t_tree, "perspective_camera_range", fPerspCamRange, fPerspCamRange);
00401          cAperture.SetValue(30.0f);
00402          GetNodeAttributeOrDefault(t_tree, "perspective_camera_aperture", cAperture, cAperture);
00403          CQuaternion cPerspCamOrient(bPerspCamFront ? CRadians::ZERO : -CRadians::PI_OVER_TWO,
00404                                      CVector3::Y);
00405          SAnchor& cPerspCamAnchor = m_pcEmbodiedEntity->AddAnchor("perspective_camera",
00406                                                                   CVector3(BODY_RADIUS, 0.0, BEACON_ELEVATION),
00407                                                                   cPerspCamOrient);
00408          m_pcPerspectiveCameraEquippedEntity =
00409             new CPerspectiveCameraEquippedEntity(this,
00410                                                  "perspective_camera_0",
00411                                                  ToRadians(cAperture),
00412                                                  fPerspCamFocalLength,
00413                                                  fPerspCamRange,
00414                                                  640, 480,
00415                                                  cPerspCamAnchor);
00416          AddComponent(*m_pcPerspectiveCameraEquippedEntity);
00417          /* Turret equipped entity */
00418          m_pcTurretEntity = new CFootBotTurretEntity(this, "turret_0", cTurretAnchor);
00419          AddComponent(*m_pcTurretEntity);
00420          /* WiFi equipped entity */
00421          m_pcWiFiEquippedEntity = new CWiFiEquippedEntity(this, "wifi_0");
00422          AddComponent(*m_pcWiFiEquippedEntity);
00423          /* Controllable entity
00424             It must be the last one, for actuators/sensors to link to composing entities correctly */
00425          m_pcControllableEntity = new CControllableEntity(this);
00426          AddComponent(*m_pcControllableEntity);
00427          m_pcControllableEntity->Init(GetNode(t_tree, "controller"));
00428          /* Update components */
00429          UpdateComponents();
00430       }
00431       catch(CARGoSException& ex) {
00432          THROW_ARGOSEXCEPTION_NESTED("Failed to initialize entity \"" << GetId() << "\".", ex);
00433       }
00434    }
00435 
00436    /****************************************/
00437    /****************************************/
00438 
00439    void CFootBotEntity::Reset() {
00440       /* Reset all components */
00441       CComposableEntity::Reset();
00442       /* Update components */
00443       UpdateComponents();
00444    }
00445 
00446    /****************************************/
00447    /****************************************/
00448 
00449 #define UPDATE(COMPONENT) if(COMPONENT->IsEnabled()) COMPONENT->Update();
00450 
00451    void CFootBotEntity::UpdateComponents() {
00452       /* Update only the components that might change */
00453       UPDATE(m_pcDistanceScannerEquippedEntity);
00454       UPDATE(m_pcTurretEntity);
00455       UPDATE(m_pcGripperEquippedEntity);
00456       UPDATE(m_pcRABEquippedEntity);
00457       UPDATE(m_pcLEDEquippedEntity);
00458    }
00459 
00460    /****************************************/
00461    /****************************************/
00462 
00463    REGISTER_ENTITY(CFootBotEntity,
00464                    "foot-bot",
00465                    "Carlo Pinciroli [ilpincy@gmail.com]",
00466                    "1.0",
00467                    "The foot-bot robot, developed in the Swarmanoid project.",
00468                    "The foot-bot is a wheeled robot developed in the Swarmanoid Project. It is a\n"
00469                    "modular robot with a rich set of sensors and actuators. For more information,\n"
00470                    "refer to the dedicated web page\n"
00471                    "(http://www.swarmanoid.org/swarmanoid_hardware.php).\n\n"
00472                    "REQUIRED XML CONFIGURATION\n\n"
00473                    "  <arena ...>\n"
00474                    "    ...\n"
00475                    "    <foot-bot id=\"fb0\">\n"
00476                    "      <body position=\"0.4,2.3,0.25\" orientation=\"45,0,0\" />\n"
00477                    "      <controller config=\"mycntrl\" />\n"
00478                    "    </foot-bot>\n"
00479                    "    ...\n"
00480                    "  </arena>\n\n"
00481                    "The 'id' attribute is necessary and must be unique among the entities. If two\n"
00482                    "entities share the same id, initialization aborts.\n"
00483                    "The 'body/position' attribute specifies the position of the bottom point of the\n"
00484                    "foot-bot in the arena. When the robot is untranslated and unrotated, the\n"
00485                    "bottom point is in the origin and it is defined as the middle point between\n"
00486                    "the two wheels on the XY plane and the lowest point of the robot on the Z\n"
00487                    "axis, that is the point where the wheels touch the floor. The attribute values\n"
00488                    "are in the X,Y,Z order.\n"
00489                    "The 'body/orientation' attribute specifies the orientation of the foot-bot. All\n"
00490                    "rotations are performed with respect to the bottom point. The order of the\n"
00491                    "angles is Z,Y,X, which means that the first number corresponds to the rotation\n"
00492                    "around the Z axis, the second around Y and the last around X. This reflects\n"
00493                    "the internal convention used in ARGoS, in which rotations are performed in\n"
00494                    "that order. Angles are expressed in degrees. When the robot is unrotated, it\n"
00495                    "is oriented along the X axis.\n"
00496                    "The 'controller/config' attribute is used to assign a controller to the\n"
00497                    "foot-bot. The value of the attribute must be set to the id of a previously\n"
00498                    "defined controller. Controllers are defined in the <controllers> XML subtree.\n\n"
00499                    "OPTIONAL XML CONFIGURATION\n\n"
00500                    "You can set the emission range of the range-and-bearing system. By default, a\n"
00501                    "message sent by a foot-bot can be received up to 3m. By using the 'rab_range'\n"
00502                    "attribute, you can change it to, i.e., 4m as follows:\n\n"
00503                    "  <arena ...>\n"
00504                    "    ...\n"
00505                    "    <foot-bot id=\"fb0\" rab_range=\"4\">\n"
00506                    "      <body position=\"0.4,2.3,0.25\" orientation=\"45,0,0\" />\n"
00507                    "      <controller config=\"mycntrl\" />\n"
00508                    "    </foot-bot>\n"
00509                    "    ...\n"
00510                    "  </arena>\n\n"
00511                    "You can also set the data sent at each time step through the range-and-bearing"
00512                    "system. By default, a message sent by a foot-bot is 10 bytes long. By using the"
00513                    "'rab_data_size' attribute, you can change it to, i.e., 20 bytes as follows:\n\n"
00514                    "  <arena ...>\n"
00515                    "    ...\n"
00516                    "    <foot-bot id=\"fb0\" rab_data_size=\"20\">\n"
00517                    "      <body position=\"0.4,2.3,0.25\" orientation=\"45,0,0\" />\n"
00518                    "      <controller config=\"mycntrl\" />\n"
00519                    "    </foot-bot>\n"
00520                    "    ...\n"
00521                    "  </arena>\n\n"
00522                    "You can also change the aperture of the omnidirectional camera. The aperture is\n"
00523                    "set to 70 degrees by default. The tip of the omnidirectional camera is placed on\n"
00524                    "top of the robot (h=0.289), and with an aperture of 70 degrees the range on the\n"
00525                    "ground is r=h*tan(aperture)=0.289*tan(70)=0.794m. To change the aperture to 80\n"
00526                    "degrees, use the 'omnidirectional_camera_aperture' as follows:\n\n"
00527                    "  <arena ...>\n"
00528                    "    ...\n"
00529                    "    <foot-bot id=\"fb0\" omnidirectional_camera_aperture=\"80\">\n"
00530                    "      <body position=\"0.4,2.3,0.25\" orientation=\"45,0,0\" />\n"
00531                    "      <controller config=\"mycntrl\" />\n"
00532                    "    </foot-bot>\n"
00533                    "    ...\n"
00534                    "  </arena>\n\n"
00535                    "Finally, you can change the parameters of the perspective camera. You can set\n"
00536                    "its direction, aperture, focal length, and range with the attributes\n"
00537                    "'perspective_camera_front', 'perspective_camera_aperture',\n"
00538                    "'perspective_camera_focal_length', and 'perspective_camera_range', respectively.\n"
00539                    "The default values are: 'true' for front direction, 30 degrees for aperture,\n"
00540                    "0.035 for focal length, and 2 meters for range. When the direction is set to\n"
00541                    "'false', the camera looks up. This can be useful to see the eye-bot LEDs. Check\n"
00542                    "the following example:\n\n"
00543                    "  <arena ...>\n"
00544                    "    ...\n"
00545                    "    <foot-bot id=\"fb0\"\n"
00546                    "              perspective_camera_front=\"false\"\n"
00547                    "              perspective_camera_aperture=\"45\"\n"
00548                    "              perspective_camera_focal_length=\"0.07\"\n"
00549                    "              perspective_camera_range=\"10\">\n"
00550                    "      <body position=\"0.4,2.3,0.25\" orientation=\"45,0,0\" />\n"
00551                    "      <controller config=\"mycntrl\" />\n"
00552                    "    </foot-bot>\n"
00553                    "    ...\n"
00554                    "  </arena>\n\n"
00555                    ,
00556                    "Under development"
00557       );
00558 
00559    /****************************************/
00560    /****************************************/
00561 
00562    REGISTER_STANDARD_SPACE_OPERATIONS_ON_COMPOSABLE(CFootBotEntity);
00563 
00564    /****************************************/
00565    /****************************************/
00566 
00567 }
```

---

Generated on 10 Jul 2018 for ARGoS by 
 1.6.1
